# K88 Fimbrial Adhesin Targeting of Microspheres Containing Gentamicin Made with Albumin Glycated with Lactose

**DOI:** 10.3390/ijms160922425

**Published:** 2015-09-16

**Authors:** Andre-i Sarabia-Sainz, Hector Manuel Sarabia-Sainz, Gabriela Ramos-Clamont Montfort, Veronica Mata-Haro, Ana María Guzman-Partida, Roberto Guzman, Mariano Garcia-Soto, Luz Vazquez-Moreno

**Affiliations:** 1 Departamento de Investigacion en Fisica, Universidad de Sonora, Hermosillo Sonora 83000, Mexico; E-Mail: andreisarabia@gmail.com; 2 Laboratorio de Bioquimica de Proteinas y Glicanos, Coordinacion de Ciencia de los Alimentos, Centro de Investigacion en Alimentacion y Desarrollo A.C., Hermosillo Sonora 83304, Mexico; E-Mails: hsarabiasainz@gmail.com (H.M.S.-S.); gramos@ciad.mx (G.R.-C.M.); vmata@ciad.mx (V.M.-H.); gupa@ciad.mx (A.M.G.-P.); 3 Departamento de Investigacion y Posgrado en Alimentos, Universidad de Sonora, Hermosillo Sonora 83000, Mexico; 4 Department of Chemical and Environmental Engineering, the University of Arizona, Tucson, AZ 85721, USA; E-Mails: guzmanr@email.arizona.edu (R.G.); mars4ward@gmail.com (M.G.-S.)

**Keywords:** lactosylated microspheres, gentamicin, targeted active drug

## Abstract

The formulation and characterization of gentamicin-loaded microspheres as a delivery system targeting enterotoxigenic *Escherichia coli* K88 (*E. coli* K88) was investigated. Glycated albumin with lactose (BSA-glucose-β (4-1) galactose) was used as the microsphere matrix (MS-Lac) and gentamicin included as the transported antibiotic. The proposed target strategy was that exposed galactoses of MS-Lac could be specifically recognized by *E. coli* K88 adhesins, and the delivery of gentamicin would inhibit bacterial growth. Lactosylated microspheres (MS-Lac1, MS-Lac2 and MS-Lac3) were obtained using a water-in-oil emulsion, containing gentamicin, followed by crosslinking with different concentrations of glutaraldehyde. Electron microscopy displayed spherical particles with a mean size of 10–17 µm. *In vitro* release of gentamicin from MS-Lac was best fitted to a first order model, and the antibacterial activity of encapsulated and free gentamicin was comparable. MS-Lac treatments were recognized by plant galactose-specific lectins from *Ricinus communis* and *Sophora japonica* and by *E. coli* K88 adhesins. Results indicate MS-Lac1, produced with 4.2 mg/mL of crosslinker, as the best treatment and that lactosylated microsphere are promising platforms to obtain an active, targeted system against *E. coli* K88 infections.

## 1. Introduction

In the last few decades, drug delivery technology has gained great interest in the pharmaceutical industry. Drug carriers, such as liposomes, nanoparticles and microspheres, are systems based in biodegradable and biocompatible polymers [1]. These systems of controlled drug delivery offer advantages over conventional methods of drug administration, namely drug protection, control delivery and patient compliance [2]. Furthermore, lipophilic drugs present better biodistribution as observed with Paclitaxel-albumin nanosuspension (Abraxane^®^), a new anticancer drug with significantly lower incidence of allergic reactions (less than 1%) and with higher response rate than its free counterpart (Taxol) [3,4]. Furthermore, several approaches have involved the use of active targeting to find pathogen or specific sites in anomalous cells to prevent damaging healthy ones [5]. For example, Langer and Farokhzad Laboratories working with drug-loaded polymeric nanoparticles coated with aptamers targeting tumor-specific cells have shown that such constructs can enhance selective killing of tumor cells [6,7].

To improve therapies, surface modification of particles with antibodies is a strategy developed to target drug delivery to certain cells that combines properties of nano- and micro-carriers with selective recognition [8]. Alternatively, the complex ligand-receptor, such as lectins and sugars, could be an appropriate approach for active drug delivery. *In vitro*, the hepatocyte asialoglycoprotein receptor recognizes a sugar ligand attached to the surface of the microspheres [9,10]. Furthermore, bovine serum albumin (BSA) microspheres grafted with galactose residues were recognized and phagocytosed by HLE human hepatoma cells [11]. Likewise, the conjugation of carbohydrates on the surface of microspheres conveyed the specific recognition of *Kluyveromyces bulgaricus*, implying that an active drug-targeting system can be implemented for the treatment of pathogenic microorganisms via lectin-sugar recognition [12].

Our group has reported that BSA chemically modified with lactose (BSA-Lac) leads to recognition of piglet pathogens, such as enterotoxigenic *Escherichia coli* K88, and by carbohydrate-binding proteins from *Ricinus communis* agglutinin I (RCA I), a galactose specific lectin [13,14]. The aim of this work was to use BSA-Lac as a polymer to obtain lactosylated microspheres (MS-Lac) that carry gentamicin and to stabilize them by crosslinking. The evaluation of antibacterial activity followed by biorecognition of galactose residues by plant lectins and microbial adhesins showed that MS-Lac could function as a potential active drug carrier for the specific treatment of *E. coli* K88 infections.

## 2. Results

**Figure 1:**
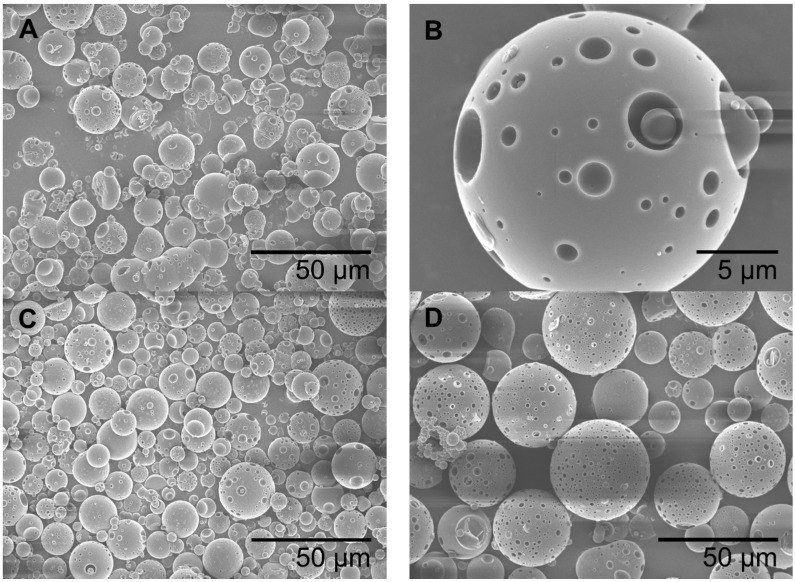
Scanning electron microscopy of albumin-lactosylated microspheres (MS-Lac). (**A**) MS-Lac1; (**B**) zoom in MS-Lac1; (**C**) MS-Lac2; (**D**) MS-Lac3.

Albumin modified with lactose (BSA-Lac) was used as the polymer for the synthesis of lactosylated albumin microspheres [14,15]. Three formulations labeled as MS-Lac1, MS-Lac2 and MS-Lac3, obtained by water-in-oil emulsion at three concentrations of crosslinker were imaged by scanning electron microscopy. All of the formulations resulted in single spherical particles (Figure 1A,C,D) with hemispherical cavities on their surface, which originated during the drying of the formulations and possibly from imprints of small aggregates that collided with the microspheres during the synthesis (Figure 1B). SEM data showed that for MS-Lac1, the polydispersity of diameter was 3 to 25 µm, for MS-Lac2 3 to 30 µm and 8 to 35 µm for MS-Lac3 (Table 1).

**Table 1:** Characteristics of albumin-lactosylated microspheres (MS-Lac) containing gentamicin.

Formulation	Glutaraldehyde in Toluene (mg/L)	Entrapment Efficiency (%) ^a^	Particle (µm) ^b^	
			Average Size	Polydispersity
MS-Lac1	4.2	89.4 ± 2.0	10	3–25
MS-Lac2	8.2	66.3 ± 2.5	12	3–30
MS-Lac3	16.8	48.5 ± 6.5	17	8–35

^a^ Average value after carrying out three independent experiments; ^b^ as obtained from SEM images.

### 2.1. Entrapment Efficiency

MS-Lac1, MS-Lac2 and MS-Lac3 presented entrapment efficiencies of gentamicin as 89.4%, 66.3% and 48.5%, respectively, as determined after dialysis studies (Table 1). Overall, the entrapment efficiency was reduced by increasing the crosslinking agent, namely by increasing the concentration of glutaraldehyde in the synthesis of the microspheres.

### 2.2. In Vitro Release Studies

**Figure 2:**
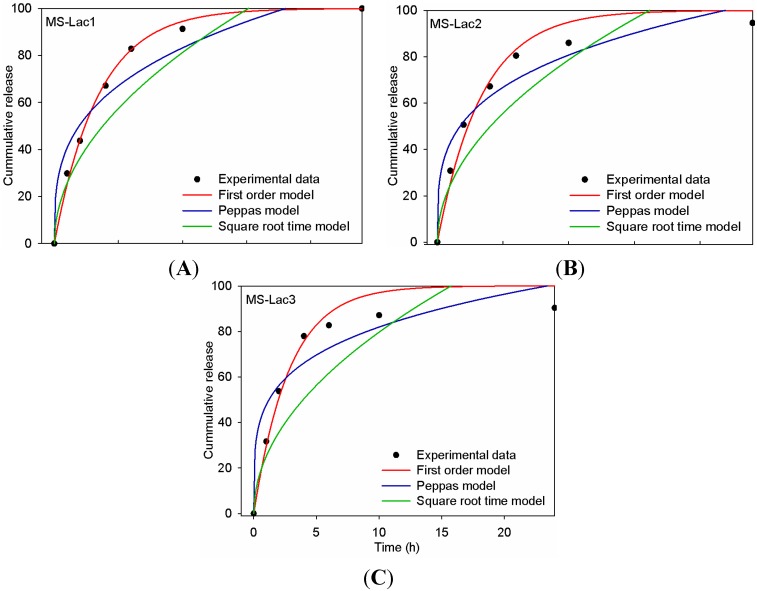
Models and experimental data matching the cumulative release of gentamicin from lactosylated microspheres (MS-Lac). (**A**) MS-Lac1; (**B**) MS-Lac2; (**C**) MS-Lac3. Data are the average of three independent release experiments.

Profiles of the cumulative release of gentamicin from MS-Lac showed that all MS-Lac treatments presented an initial burst in the first four hours followed by a slow release (Figure 2). The crosslinking conditions generated slight changes in the release profile. Furthermore, MS-Lac1, MS-Lac2 and MS-Lac3 experimental data of the release of gentamicin were 98%, 94% and 90%, respectively, after 24 h.

To correlate the drug release *in vitro* and *in vivo*, the mechanisms governing the antibiotic release from lactosylated microspheres were determined [16]. Figure 2 shows the experimental data of gentamicin released from lactosylated microspheres compared to first order, Peppas and square root time models. The data of the *in vitro* drug release from microspheres was fitted to find the parameters of these models, finding *r*^2^, *k* and *n* as shown in Table 2. These results indicate that the best adjusted model was the first order with an *r*^2^ of 0.965, 0.973 and 0.960 for MS-Lac1, MS-Lac2 and MS-Lac3, respectively. As reported elsewhere, this model is applicable to the dissolution of drugs from porous matrixes, such as albumin microspheres [17]. The diffusional exponent (*n*) of the Peppas model for all treatments was less than 0.43, thus indicating that the mechanism followed a Fickian diffusion process [18].

**Table 2:** Parameters describing the release of gentamicin from lactosylated microspheres (MS-Lac) in PBS (pH 7.2).

Formulation	First Order *F* = 1 − *e*^(−*k*∙*t*)^		Peppas *F* = *k*∙*t^n^*		Square root time *F* = *k*∙*t*^2^	
	*r* ^2^	*k*	*r* ^2^	*n*	*r* ^2^	*k*
MS-Lac1	0.97	0.352	0.91	0.234	0.68	25.19
MS-Lac2	0.97	0.297	0.95	0.273	0.74	24.87
MS-Lac3	0.96	0.353	0.91	0.2341	0.61	25.19

### 2.3. In Vitro Antimicrobial Activity

*E. coli* K88 was used as a model to test the antimicrobial activity of the gentamicin released from MS-Lac. Minimal inhibitory concentrations (MIC) of MS-Lac1, MS-Lac2 and MS-Lac3 were 2.16, 2.92 and 4 µg/mL, respectively; with each of these treatments containing a load of 0.31 µg/mL of gentamicin as determined by EE (Table 3). Ms-Lac1 was more effective, since less mass was required to reach the MIC. It is important to point out that, as expected, MICs of free gentamicin and equivalent gentamicin in microspheres were identical.

**Table 3:** Minimal inhibitory concentration (MIC) of gentamicin released from lactosylated microspheres (MS-Lac).

	MIC (µg/mL)	
Formulation	MS-Lac	Estimated Gentamicin in Microspheres *
MS-Lac1	2.16	0.31
MS-Lac2	2.92	0.31
MS-Lac3	4.00	0.31

* According to entrapment efficiency data. MIC of free gentamicin = 0.31 µg/mL.

### 2.4. Interaction Assays

**Figure 3:**
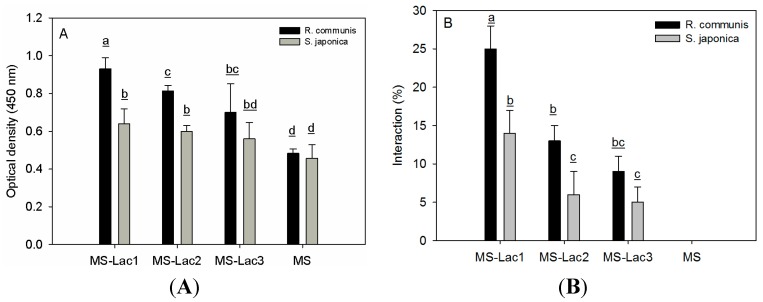
Assay of the recognition of *Ricinus communis* and *Sophora japonica* lectins for three treatments of lactosylated (MS-Lac) and non-lactosylated (MS) albumin microspheres. (**A**) Enzyme-linked lectin recognition assay (ELLA); (**B**) flow cytometry assay. Data are the average of three independent experiments and analyzed by Tukey’s test; *p* ≤ 0.05. Means with the same letter are not significantly different.

**Figure 4:**
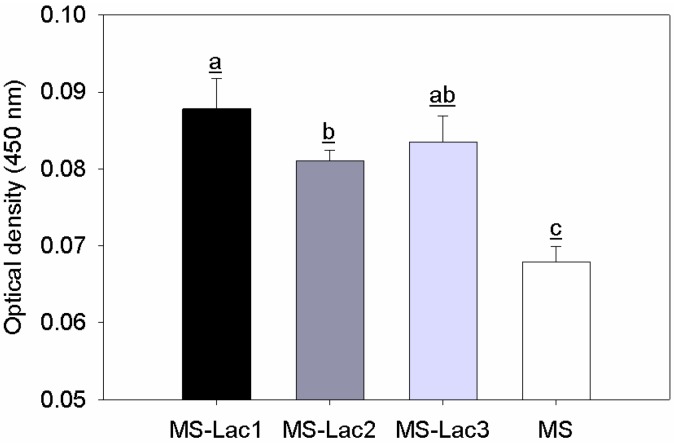
Recognition assay of partially-purified K88 adhesin for lactosylated (MS-Lac) and non-lactosylated (MS) albumin microspheres using ELLA assays. Experiments were performed in triplicate and evaluated by analysis of variance (ANOVA) followed by the application of Tukey’s test; *p* ≤ 0.05. Means (a, b, ab and c, respectively) with the same letter are not significantly different.

Biorecognition of the galactose residues linked to MS-Lac were evaluated using carbohydrate-binding proteins by ELLA and flow cytometry, shown in Figure 3A,B, respectively. In addition, the interaction was accomplished using partially-purified microbial K88 adhesins (Figure 4). The interaction of MS-Lac with galactose-specific lectins included RCA1 and SJA and microspheres (MS) prepared with unmodified BSA as the control. Plant lectin recognition showed statistical differences (*p* < 0.05), particularly with MS-Lac1 and MS-Lac2, when compared to unmodified MS (Figure 3A). Given the specificity for both lectins, RCA1 presented a higher interaction than SJA. Furthermore, both lectins showed non-specific binding to non-lactosylated microspheres (MS), probably due to the hydrophobic interaction commonly observed with albumin.

Similar results to ELLA data were found by flow cytometry (Figure 3B). RCA1 and SJA displayed an interaction with treatments in the following order: MS-Lac1 > MS-Lac2 > MS-Lac3. As expected, other non-galactose-specific lectins incorporated as controls (Con A and VVA) did not reveal interactions with lactosylated microspheres (data not shown). For both types of assay (ELLA and flow cytometry), the recognition was higher with less crosslinking, indicating that the availability of carbohydrate is necessary for the lectin-sugar interaction; MA-Lac1 appeared to have more galactose exposed for biorecognition than MS-Lac2 and MS-Lac3, even though they all were prepared using the same stock of lactosylated albumin.

K88 adhesins presented a higher recognition for all lactosylated microspheres than for non-lactosylated microspheres. The interaction displayed no significant difference (*p* > 0.05) between MS-Lac1 and MS-Lac3 or MS-Lac2 and MS-Lac3. However, a statistical difference (*p* < 0.05) was observed between MS-Lac1 and MS-Lac2 and the control (Figure 4).

## 3. Discussion

Enterotoxigenic *Escherichia coli* (ETEC) is a major cause of illness and death in neonatal and, recently, weaned pigs. K88 strains of ETEC that cause diarrhea in pigs possess an adhesin essential for disease to occur [19]. Recent reports show that K88 adhesin targets specific receptors containing galactose exposed when using lactosylated albumin [13,14]. In this study, the formulation of stabilized microspheres, antibiotic carriers, using lactosylated albumin as encapsulation polymer, was examined.

Albumin offers several advantages, is a natural, biodegradable and biocompatible polymer, forms stable microspheres and clinically is widely used as a drug carrier [20]. In a previous report, we obtained albumin microcapsules crosslinked with glutaraldehyde that were resistant to conditions of pH and enzyme activity similar to those found in the early weaned piglet digestive tract [21].

This study results showed that the amount of crosslinker had no apparent effect on the MS-Lac microspheres’ morphology; however, a slight increase in the diameter was observed with greater concentration (Figure 1). All MS-Lac treatments show a size larger than 10 µm, a dimension expected to prevent intestinal absorption, allowing this to remain in the gut [22]. The particle size plays an important role in the deposition and localization of microspheres in the body [23], as the retention of microspheres in the intestine would play an important role for therapy against *E. coli* K88 infecting susceptible piglets [19].

On the contrary, the crosslinking condition affected the entrapment efficiency, which was reduced by increasing the concentration of glutaraldehyde during the synthesis (Table 1). This effect could be attributed to the binding of gentamicin, through its amino groups, leading to incomplete release of antibiotic, as has been reported previously with other drugs containing primary amino groups [24]. Results were also consistent with other reports that attributed to crosslinking more rigid particles with a decrease in the pores size, which ultimately can affect the release of drugs [25].

The mathematical models applied for describing the kinetics of the drug release process from microspheres showed that gentamicin release could be better described by a first order equation. This indicates the transport of drug by a concentration gradient [26]. It is important to point out that for all of the MS-Lac particles, 50% of gentamicin was released just after 3 h, providing advantages for the treatment of pig intestinal infections, which, during diarrhea, the transit from intake to gastric emptying may take from 3 to 4 h [27].

Additionally, our results support the concept that the process used for the synthesis of gentamicin loaded in microspheres does not decrease the antibacterial activity, and consequently, the MICs were preserved, contrary to observations when high temperature or other drastic conditions are applied [28,29].

Synthesized MS-Lac microspheres were recognized by galactose-specific carbohydrate-binding proteins (lectins) and microbial adhesin (K88 adhesin), consistent with the exposure of galactoses [30,31]. Furthermore, both lectins showed a decrease in recognition with increased crosslinking, implying that the availability of galactose is reduced. Studies have shown that crosslinking alters the antibody to antigen interaction due to the obstruction of recognition sites [32]. Furthermore, K88 adhesins binding to MS-Lac treatments were comparable to plant lectins, where MS-Lac1 exhibited greater biorecognition.

Bacterial adhesins’ affinity to lactosylated microspheres is of great interest, because of the potential applications to the different classes of diarrheagenic *E. coli* (enterotoxigenic, enteropathogenic, enterohemorrhagic, enteroinvasive and enteroaggregative) and parasites, the first step of which is infection, mediated by carbohydrate binding proteins [33].

## 4. Experimental

### 4.1. Materials

BSA Fraction V, gentamicin sulfate, glutaraldehyde and Span^®^ 80 were purchased from Sigma-Aldrich, (St Louis, MO, USA). Biotin-labeled lectins were from Vector (Burlingame, CA, USA). All other chemicals used were analytical grade. BSA glycation (BSA lactosylation) was conducted according to Sarabia-Sainz *et al.* [10]. Enterotoxigenic *E. coli* K88 was donated by Dr. Carlos Eslava from the Universidad Nacional Autonoma de Mexico.

### 4.2. Preparation of Microspheres

Lactosylated albumin microspheres loaded with gentamicin (MS-Lac) were prepared according to Mathew *et al.*, 2007 [15]. Briefly, 1 mL of aqueous solution containing 20% BSA-Lac and 0.4% gentamicin sulfate were added to 30 mL of mineral oil including 0.1% (wt/wt) Span^®^ 80 under continuous stirring at 2000 rpm for 1 h to obtain a homogeneous emulsion, followed by the addition of 4.2, 8.2 and 16.8 mg/mL of glutaraldehyde saturated with toluene to crosslink MS-Lac1, MS-Lac2 and MS-Lac3 microspheres, respectively. Four hours later, the microspheres were recovered by centrifugation (5 min at 300× *g*), washed three times with hexane and dried at room temperature.

### 4.3. Morphology Studies

The morphology was examined by scanning electron microscopy (SEM) using a Hitachi S-4800 FE-SEM operating at 5 kV. Samples were immobilized on carbon tape and sputtered with gold/palladium (60/40) to provide a conductive coating of 2 nm prior to imaging [15].

### 4.4. Entrapment Efficiency

Fifty milligrams of each MS-Lac was dispersed in 5 mL of PBS (pH 7.2), placed in a dialysis bag (12,000 MWCO) and submerged in 30 mL of PBS (pH 7.2). Later, the suspension of microspheres was placed in a shaking bath for 72 h [28]. The concentration of gentamicin was quantified photometrically at 320 nm after derivatization with O-phtaldehyde [34]. The O-phtaldehyde agent was prepared as described [35]. Sixty milligrams of O-phtaldehyde were solubilized in 30 mL of 0.1 M sodium borate with the addition of 200 µL of 2-mercaptoethanol and 20 mL of distilled water. The entrapment efficiency (EE) was calculated using the following formula:

$$\mathrm{EE}\;\left(\%\right)=\frac{\mathrm{amount}\;\mathrm{of}\;\mathrm{gentamicin}\;\mathrm{found}\;\mathrm{in}\;\mathrm{microspheres}\times 100}{\mathrm{amount}\;\mathrm{of}\;\mathrm{gentamicin}\;\mathrm{used}}$$

### 4.5. In Vitro Release Studies

Twenty milligrams of microspheres were placed in a dialysis bag containing 2 mL of PBS (pH 7.2) and suspended in 20 mL PBS (pH 7.2). Dialysis was carried out at 37 °C with continuous agitation. At specific time intervals, two milliliters of the external phase were taken to quantify the released gentamicin as described before. Two milliliters of fresh buffer were added to the external phase to compensate for the volume removed. First order [36], Peppas [37] and square root time [38] models were explored to find the best fit of release over time in order to describe the drug release from the microspheres. The release parameters were calculated with SigmaPlot 11.

### 4.6. In Vitro Antimicrobial Activity

*E. coli* strain K88 kinetics of growth were determined by the microtiter broth method [39,40]. The load of gentamicin in MS-Lac was estimated and adjusted to 20 µg/mL as the initial concentration. Suspensions were stirred constantly and serially diluted in a nutrient broth containing from 20 to 0.31 µg/mL of gentamicin. Aliquots of 200 µL of each suspension of MS-Lac containing gentamicin were transferred to a sterile microplate. Each dilution was incubated with 3 µL of *E. coli* K88 in suspension (10^8^ CFU·mL^−1^). Bacterial growth was estimated using a microplate reader (optical density 620 nm, ELISA 680 Anthos Zenyth 340, Cambridge, UK). Plate incubation was performed at 37 °C for 24 h with readings at 30-min intervals. Untreated *E. coli* K88 was used as a positive growth control.

### 4.7. Enzyme-Linked Lectin Recognition Assays

Recognition of carbohydrates attached to MS-Lac was done by biotin-labeled *Ricinus communis I* lectin, RCA1, and *Sophora japonica* lectin, SJA [14]. Polystyrene ELISA microtiter plates were coated with 100 μL of 0.5 mg/mL MS-Lac in 50 mM sodium bicarbonate buffer (pH 9.5) and adsorbed for 16 h at 4 °C. Following adsorption, the wells were blocked for 3 h with 1.5% BSA dissolved in 20 mM PBS containing 0.05% Tween 20 (TPBS, pH 7.5). Aliquots (100 μL) of biotinylated lectin (5 μg/mL) in PBS (pH 7.5) were added and incubated for 2 h. After washing with TPBS, the plates were incubated with avidin-peroxidase (1/1000 *v*/*v* in PBS pH 7.5) for 1 h, and O-phenylendiamine was used to reveal the binding of lectin. The solutions absorbance was measured using a microplate reader (ELISA 680 Anthos Zenyth 340, Cambridge, UK) at 450 nm.

### 4.8. Enzyme-Linked Adhesin Recognition Assays

Adhesins isolation from *E. coli* K88 was achieved from strains grown in blood agar plates for 18 h at 37 °C. The cells scraped off from these surfaces were suspended in 15 mL of 50 mM Tris-HCl (pH 7.4) containing 1 M NaCl and disrupted using an Ultra Turrax T25 (Janke-Kunkel IKA-Labortechnik, Lima, OH, USA) at 24,000 rpm during 10 min. Large cell fragments were removed by two consecutive centrifugations for 10 min at 10,000 and 14,000× *g*, respectively. Next, the supernatants were saturated with 60% (NH_4_)_2_SO_4_. Finally, partially-purified K88 adhesins were obtained by centrifugation at 14,000× *g* for 20 min, extensively dialyzed and stored at −40 °C until use [41].

Recognition of partially-purified K88 adhesin to MS-Lac was conducted as described for enzyme-linked lectin recognition assays (ELLA). Briefly, ELISA plates were coated with 100 μL of 0.5 mg/mL MS-Lac in 50 mM sodium bicarbonate buffer (pH 9.5) and incubated overnight at 4 °C. After extensive washes, the wells were blocked for 3 h with 1.5% BSA in TPBS. One hundred microliters of biotin-labeled K88 adhesin (1 mg/mL) were added and incubated for 2 h. After a single wash with TPBS, the plates were treated with avidin-peroxidase (1/1000 *v*/*v* in PBS, pH 7.5) for 1 h and the interaction revealed by the use of O-phenylendiamine as described before.

### 4.9. Flow Cytometry

Interaction assays of MS-Lac with lectins were conducted by flow cytometry [42]. Briefly, aliquots of 1 mg/mL MS-Lac were incubated with biotinylated galactose-specific lectins, RCA I and SJA (5 μg/mL) at 20 °C for 40 min. MS-Lac preparations were then washed 3 times with TPBS and incubated with FITC-conjugated streptavidin at 20 °C for 40 min. Ms-Lac-streptavidin-FITC conjugated to the surface was incubated with lectins and K88 adhesin and analyzed using a BD FACSCanto II (BD biosciences, San Jose, CA, USA) flow cytometer. Controls included non-galactose specific lectins, *Canavalia ensiformis* (Con A, mannose specific) and *Vicia villosa* (VVA, α-GalNac specific), as well as non-lactosylated microspheres (MS). At least 10,000 microspheres were acquired from each sample, and FITC-stained microspheres were taken as positive events. The experiments were performed in triplicate and evaluated by analysis of variance (ANOVA) followed by the application of the Tukey test with *p* ≤ 0.05.

## 5. Conclusions

Microspheres modified with carbohydrates are promising platforms to promote biorecognition for bacteria that cause infections mediated by sugar binding adhesins.

## References

[1] Sinha V.R., Aggarwal A., Trehan A. (2004). Biodegradable pegylated microspheres and nanospheres. Am. J. Drug Deliv..

[2] Varde N.K., Pack D.W. (2004). Microspheres for controlled release drug delivery. Expert Opin. Biol. Ther..

[3] Wang A.Z., Langer R., Farokhzad O.C. (2012). Nanoparticle delivery of cancer drugs. Annu. Rev. Med..

[4] Goldstein D., El-Maraghi R.H., Hammel P., Heinemann V., Kunzmann V., Sastre J., Scheithauer W., Siena S., Tabernero J., Teixeira L. (2015). Nab-paclitaxel plus gemcitabine for metastatic pancreatic cancer: Long-term survival from a phase iii trial. J. Natl. Cancer Inst..

[5] Langer K., Anhorn M., Steinhauser I., Dreis S., Celebi D., Schrickel N., Faust S., Vogel V. (2008). Human serum albumin (hsa) nanoparticles: Reproducibility of preparation process and kinetics of enzymatic degradation. Int. J. Pharm..

[6] Dhar S., Kolishetti N., Lippard S.J., Farokhzad O.C. (2011). Targeted delivery of a cisplatin prodrug for safer and more effective prostate cancer therapy *in vivo*. Proc. Natl. Acad. Sci. USA.

[7] Oh S.S., Lee B.F., Leibfarth F.A., Eisenstein M., Robb M.J., Lynd N.A., Hawker C.J., Soh H.T. (2014). Synthetic aptamer-polymer hybrid constructs for programmed drug delivery into specific target cells. J. Am. Chem. Soc..

[8] Arruebo M., Valladares M., González-Fernández Á (2009). Antibody-conjugated nanoparticles for biomedical applications. J. Nanomater..

[9] Zhang C., Cheng Y., Qu G., Wu X., Ding Y., Cheng Z., Yu L., Ping Q. (2008). Preparation and characterization of galactosylated chitosan coated bsa microspheres containing 5-fluorouracil. Carbohydr. Polym..

[10] Yang Y., Yuan S.-X., Zhao L.-H., Wang C.-W., Ni J.-S., Wang Z.-G., Lin C., Wu M.-C., Zhou W.-P. (2015). Ligand-directed stearic acid grafted chitosan micelles to increase therapeutic efficacy in hepatic cancer. Mol. Pharm..

[11] Ohya Y., Takei T., Fukushima H., Ouchi T. (1991). Preparation of albumin microspheres grafted galactose residues through polyethylene-glycol spacers, release behavior of 5-fluorouracil from them, and their lectin-mediated aggregation. J. Macromol. Sci. Chem..

[12] Kassab R., Parrot-Lopez H., Fessi H., Menaucourt J., Bonaly R., Coulon J. (2002). Molecular recognition by kluyveromyces of amphotericin b-loaded, galactose-tagged, poly (lactic acid) microspheres. Bioorg. Med. Chem..

[13] Ledesma-Osuna A.I., Ramos-Clamont G., Vazquez-Moreno L. (2009). Biorecognition of chemically modified bovine serum albumin with lactose prepared under different conditions. J. Agric. Food Chem..

[14] Sarabia-Sainz A.-I., Ramos-Clamont G., Winzerling J., Vázquez-Moreno L. (2011). Bacterial recognition of thermal glycation products derived from porcine serum albumin with lactose. Acta Biochim. Pol..

[15] Mathew S.T., Devi S.G., Sandhya K. (2007). Formulation and evaluation of ketorolac tromethamine-loaded albumin microspheres for potential intramuscular administration. Aaps Pharmscitech.

[16] Zahirul M., Khan I. (1996). Dissolution testing for sustained or controlled release oral dosage forms and correlation with *in vivo* data: Challenges and opportunities. Int. J. Pharm..

[17] Narasimhan B., Mallapragada S., Peppas N. (1999). Release kinetics, data interpretation. Encycl. Control. Drug Deliv..

[18] Costa P., Sousa Lobo J.M. (2001). Modeling and comparison of dissolution profiles. Eur. J. Pharm. Sci..

[19] Grange P.A., Mouricout M.l.A., Levery S.B., Francis D.H., Erickson A.K. (2002). Evaluation of receptor binding specificity of escherichia coli k88 (f4) fimbrial adhesin variants using porcine serum transferrin and glycosphingolipids as model receptors. Infect. Immun..

[20] Yan F., Li B., Shen F., Fu Q. (2014). Formulation and characterization of albumin microspheres containing norcantharidate for liver tumor targeting. Drug Deliv..

[21] Sarabia-Sainz A.-I., Montfort G.R.-C., Lizardi-Mendoza J., Sáchez-Saavedra M.D.P., Candia-Plata M.D.C., Guzman R.Z., Lucero-Acuña A., Vazquez-Moreno L. (2012). Formulation and characterization of gentamicin-loaded albumin microspheres as a potential drug carrier for the treatment of e. Coli k88 infections. Int. J. Drug Deliv..

[22] Damgé C., Maincent P., Ubrich N. (2007). Oral delivery of insulin associated to polymeric nanoparticles in diabetic rats. J. Control. Release.

[23] Patil G.V. (2003). Biopolymer albumin for diagnosis and in drug delivery. Drug Dev. Res..

[24] Leo E., Angela Vandelli M., Cameroni R., Forni F. (1997). Doxorubicin-loaded gelatin nanoparticles stabilized by glutaraldehyde: Involvement of the drug in the cross-linking process. Int. J. Pharm..

[25] Thakkar H.P., Murthy R.R. (2008). Effect of cross-linking agent on the characteristics of celecoxib loaded chitosan microspheres. Asian J. Pharm..

[26] Peppas N. 1. (2014). Commentary on an exponential model for the analysis of drug delivery: Original research article: A simple equation for description of solute release: I II. Fickian and non-fickian release from non-swellable devices in the form of slabs, spheres, cylinders or discs, 1987. J. Control. Release: Off. J. Control. Release Soc..

[27] Theodorou V., Fioramonti J., Hachet T., Bueno L. (1991). Absorptive and motor components of the antidiarrhoeal action of loperamide: An *in vivo* study in pigs. Gut.

[28] Haswani D.K., Nettey H., Oettinger C., D’Souza M.J. (2006). Formulation, characterization and pharmacokinetic evaluation of gentamicin sulphate loaded albumin microspheres. J. Microencapsul..

[29] Stubbings W., Bostock J., Ingham E., Chopra I. (2006). Mechanisms of the post-antibiotic effects induced by rifampicin and gentamicin in escherichia coli. J. Antimicrob. Chemother..

[30] Sarabia-Sainz A.-I., Ramos-Clamont G., Candia-Plata M.D.C., Vázquez-Moreno L. (2009). Biorecognition of *escherichia coli* k88 adhesin for glycated porcine albumin. Int. J. Biol. Macromol..

[31] Myllyharju J., Nokkala S. (1998). Localization and identification of galactose/n-acetylgalactosamine and sialic acid-containing proteins in chinese hamster metaphase chromosomes. Cell Biol. Int..

[32] Moreno N., Chevalier M., Ronzon F., Manin C., Dupuy M., Krell T., Rieu J.P. (2011). Unbinding forces of single pertussis toxin-antibody complexes measured by atomic force spectroscopy correlate with their dissociation rates determined by surface plasmon resonance. J. Mol. Recognit..

[33] Vandana Grover S.G., Chakraborti Anuradha, Majumdar Siddhartha, Ganguly Nirmal Kumar (2007). Galactose-specific fimbrial adhesin of enteroaggregative escherichia coli: A possible aggregative factor. Curr. Microbiol..

[34] Prior S., Gander B., Lecároz C., Irache J.M., Gamazo C. (2004). Gentamicin-loaded microspheres for reducing the intracellular brucella abortus load in infected monocytes. J. Antimicrob. Chemother..

[35] Fayle S.N.E., Healy J.P., Brown P.A., Reid E.A., Gerrard J.A., Ames J.M. (2001). Novel approaches to the analysis of the maillard reaction of proteins. Electrophoresis.

[36] Shah M.V., de Gennaro M.D., Suryakasuma H. (1987). An evaluation of albumin microcapsules prepared using a multiple emulsion technique. J. Microencapsul..

[37] Peppas N. (1984). Analysis of fickian and non-fickian drug release from polymers. Pharm. Acta Helv..

[38] Higuchi T. (1961). Rate of release of medicaments from ointment bases containing drugs in suspension. J. Pharm. Sci..

[39] Holowachuk S.A., Bal’a M.F., Buddington R.K. (2003). A kinetic microplate method for quantifying the antibacterial properties of biological fluids. J. Microbiol. Methods.

[40] Lourenço F.R., Pinto T.J.A. (2011). Antibiotic microbial assay using kinetic-reading microplate system. Braz. J. Pharm. Sci..

[41] Mooi F., de Graaf F., van Embden J. (1979). Cloning mapping and expression of the genetic determinant that encodes for the k88ab antigen. Nucleic Acids Res..

[42] Keegan M.E., Royce S.M., Fahmy T., Saltzman W.M. (2006). *In vitro* evaluation of biodegradable microspheres with surface-bound ligands. J. Control. Release.

